# A 3-month music-based recreation program added to stable speech-language therapy in institutionalized patients with Parkinson's disease: a feasibility study

**DOI:** 10.1016/j.prdoa.2026.100485

**Published:** 2026-07-30

**Authors:** Yasufumi Oi, Haruna Aoki, Tomoyuki Hatsuse, Seira Taniguchi, Seiji Ueno, Akiyoshi Yamamoto, Kensuke Ikenaka

**Affiliations:** a Super Court Co., Ltd., Osaka, Japan; b Shizuku Works, Toyonaka, Japan; c Shion Hospital, Medical Corporation Yoshikenkai, Osaka, Japan; d Department of Neurology, Graduate School of Medicine, The University of Osaka, Suita, Japan

**Keywords:** Parkinson's disease, Dysphagia, Dysphonia, Music therapy, Voice rehabilitation

## Abstract

Dysphonia and dysphagia are highly prevalent in late-stage Parkinson's disease (PD), particularly among patients residing in long-term care facilities, yet evidence for rehabilitation interventions in this population remains extremely limited. With increasing longevity, more patients progress to advanced stages marked by frailty, long disease duration, and treatment-resistant symptoms, making the development of effective therapeutic strategies an urgent clinical priority. Here, we conducted an uncontrolled, single-arm, open-label exploratory pre–post feasibility study to evaluate a 12-session group music recreation program added to stable ongoing speech-language therapy in institutionalized patients with Parkinson's disease. Twenty-five of 32 enrolled patients attended at least three of the 12 scheduled sessions and completed pre- and post-program assessments. Outcomes included Maximum Phonation Time (MPT), mean sound pressure, Vocal Handicap Index (VHI), modified water swallowing test (MWST), repetitive saliva swallowing test, Dysphagia Severity Scale, swallowing visual analog scale, and a dysphagia screening questionnaire. In unadjusted Wilcoxon signed-rank analyses, nominal pre–post changes were observed in MPT and MWST, and VHI showed a non-significant trend toward reduction; however, no outcome remained statistically significant after Bonferroni correction. Other swallowing measures and subjective swallowing outcomes did not show clear improvement. These findings suggest that adding group music recreation to stable speech-language therapy is feasible in institutionalized patients with PD and may provide hypothesis-generating signals for selected voice and structured water-swallowing outcomes; controlled studies are needed to determine efficacy.

## Highlights

• Evidence for rehabilitation in institutionalized advanced PD is scarce.
• Music-based program combined group activities and vocal training.
• Phonatory performance improved after the 3-month intervention.
• Swallowing improvement was limited to task-specific objective measures.
• Objective swallowing gains did not match subjective dysphagia perception.

## 1 Introduction

Dysphonia and dysphagia are common and clinically significant complications of Parkinson's disease (PD), contributing to aspiration risk, malnutrition, reduced quality of life, and increased institutional care burden. In advanced stages of PD, particularly among institutionalized individuals, dysphagia is often multifactorial, involving impaired respiratory–phonatory coordination, reduced laryngeal closure, bradykinesia of swallowing musculature, and diminished sensory feedback.

Intensive individualized speech therapies, including high-effort voice treatment approaches such as Lee Silverman Voice Treatment (LSVT-LOUD), have demonstrated efficacy in improving vocal amplitude and speech intelligibility in ambulatory PD populations [1], [2]. Some studies have suggested that high-effort phonatory training may also influence swallowing function through shared laryngeal and respiratory mechanisms [2], [3], [4]. However, implementation of such structured interventions in long-term care settings is frequently limited by logistical constraints and patient frailty.

Group-based music interventions may enhance engagement while stimulating respiratory and vocal effort, potentially influencing swallowing physiology through overlapping neuromuscular pathways. In the present program, music-based activities were designed to include respiratory muscle stretching, loud reading of song lyrics to promote vocal projection, and singing of familiar songs to facilitate sustained phonation and coordinated breathing. Nevertheless, evidence regarding the feasibility of adding structured group-based music activities to stable background speech-language therapy in institutionalized PD populations remains scarce. While our previous investigations have focused on clinical and biological aspects of Parkinson's disease [5] [6] [7], structured rehabilitation approaches targeting advanced institutionalized patients have not been sufficiently examined. We therefore examined the feasibility of adding a 12-session group music recreation program to stable ongoing speech-language therapy in institutionalized patients with PD and explored pre–post changes in multidimensional voice and swallowing outcomes.

## 2 Materials and methods

### 2.1 Study design

This was an uncontrolled, single-arm, open-label, exploratory pre–post feasibility study conducted in a long-term care setting. The study evaluated the feasibility of adding a 12-session group music recreation program to stable ongoing speech-language therapy in institutionalized patients with Parkinson's disease. Voice and swallowing outcomes were assessed before and after the 3-month program as exploratory clinical outcomes.

### 2.2 Background speech-language therapy

All participants had been receiving speech-language therapy as part of routine care before the start of the music recreation program. Briefly, individually delivered high-effort vocal exercises were administered by a speech therapist certified in LSVT-LOUD. The full standardized LSVT-LOUD protocol was not applied; instead, a simplified and adapted approach focusing on increased vocal effort, sustained phonation, and respiratory–phonatory coordination was implemented in one-on-one sessions tailored to each participant's functional level. The frequency and content of speech-language therapy were kept unchanged throughout the 3-month study period.

### 2.3 Participants

Patients were eligible to participate if they were institutionalized residents who met the Movement Disorder Society (MDS) clinical diagnostic criteria for Parkinson's disease, were receiving stable speech-language therapy, and were able to participate in group music recreation sessions. Participants were not required to meet predefined diagnostic thresholds for both dysphonia and dysphagia. Thirty-two patients consented to participate in the 12-session group music recreation program. Participants were included in the final analysis if they attended at least three of the 12 scheduled music recreation sessions and completed both pre- and post-program assessments. Of the 32 consenting participants, 25 met these criteria and were included in the final analysis.

### 2.4 Ethics

The protocol conformed to Helsinki Declaration principles and was approved by the Osaka University review board (approval numbers 13,471 and 22,311). All participants provided written informed consent before any assessment, including the collection of demographic information such as age and sex.

### 2.5 Group music recreation program

The intervention of interest was a group music recreation program consisting of 12 weekly sessions over 3 months. Each session lasted 45 min and was conducted in a common room within the institution by a music therapist. Attendance was recorded at each session. One session was defined as one scheduled group music recreation session.

The overall structure of each session remained consistent across all sessions. Each session consisted of the following components:

(A) stretching (10 min), aimed at relaxing muscles involved in phonation and respiration to facilitate subsequent activities; (B) vocalization exercises (10 min), including repetitive articulation tasks (e.g., “pa–ta–ka–ra”) performed with clear and loud pronunciation to enhance basic speech and voice production; (C) reading of song lyrics (5 min), to promote attention to verbal content and prepare participants for vocal expression; (D) singing (10 min), involving one consistent theme song used across sessions and one familiar song selected for each session, both performed with emphasis on loud and clear vocalization; and (E) singing combined with coordinated movements (10 min), designed as a dual-task activity to promote cognitive–motor integration. The program used simple, widely recognized songs familiar to older adults.

All participants had been receiving speech-language therapy as part of routine care before the start of the music recreation program. Briefly, individually delivered high-effort vocal exercises were administered by a speech therapist certified in LSVT-LOUD. The full standardized LSVT-LOUD protocol was not applied; instead, a simplified and adapted approach focusing on increased vocal effort, sustained phonation, and respiratory–phonatory coordination was implemented in one-on-one sessions tailored to each participant's functional level. The frequency and content of speech-language therapy were kept unchanged throughout the 3-month study period. Therefore, the intervention of interest in this study was the addition of the group music recreation program to stable background speech-language therapy.

### 2.6 Outcome measures

Voice-related outcomes included Maximum Phonation Time (MPT) [8], mean sound pressure (MSP) [9], and the total Vocal Handicap Index (VHI) [10].

Swallowing outcomes included the modified water swallowing test (MWST) [11], repetitive saliva swallowing test (RSST) [11], Dysphagia Severity Scale (DSS), a Visual Analog Scale (VAS) for perceived swallowing difficulty, and a Seirei Dysphagia Questionnaire [11]. The Seirei Dysphagia Questionnaire is a 15-item symptom-based dysphagia screening questionnaire assessing swallowing-related symptoms. Higher scores indicate greater dysphagia-related symptom burden (Range: 0 to 60). DSS was graded using the Fujishima Dysphagia Severity Scale, a 7-level clinical classification based on actual oral intake status (1 = no oral intake; 7 = normal diet without restriction) [12]. Assessments were performed immediately before initiation and immediately after completion of the 3-month program. Assessments were scheduled during each participant's usual clinically defined ON period based on their routine antiparkinsonian medication schedule and daily ON/OFF fluctuations. Before each assessment, a rehabilitation therapist clinically confirmed that the participant was in the ON state, based on the absence of overt wearing-off or OFF symptoms. Outcome assessors were not blinded to the intervention period, and some assessments were conducted by personnel involved in routine clinical care.

### 2.7 Statistics

Statistical analyses were performed using SPSS version 29 (IBM Corp., Armonk, NY, USA). Because of the small sample size and the ordinal nature of several outcomes, pre–post comparisons were performed using the Wilcoxon signed-rank test. Continuous and ordinal variables are presented as median [interquartile range]. To account for multiple exploratory outcome comparisons, Bonferroni correction was applied across the eight pre–post outcome tests. Bonferroni-adjusted *p* values were calculated by multiplying each unadjusted p value by eight, with values greater than 1.00 set to 1.00. Effect size r was calculated as |Z|/√N. Median paired changes were calculated as post-program values minus pre-program values. Confidence intervals for median paired changes were estimated using bootstrap resampling with 1000 iterations. Two-sided *p* values are reported.

## 3 Results

### 3.1 Feasibility and attendance

Thirty-two patients consented to participate in the 12-session group music recreation program. Among all 32 consenting participants, the median number of attended sessions was 8 [IQR, 3–11.75; range, 0–12] out of 12 scheduled sessions. Twenty-five participants attended at least three sessions and completed both pre- and post-program assessments; these participants were included in the final analysis. The attendance-threshold completion rate was 78.1% (25/32). Among the 25 analyzed participants, the median number of attended sessions was 10 [IQR, 6.5–12.0; range, 3–12].

The median age of the analyzed participants was 80.0 years [IQR, 73.0–86.0], and 12 participants were female (48%). The median disease duration was 11.0 years [IQR, 5.0–15.0], the median Hoehn and Yahr stage was 4.0 [IQR, 3.0–4.5], and the median MDS-UPDRS Part III score was 46.0 [IQR, 22.0–54.0]. No changes were made to antiparkinsonian medication regimens during the 3-month study period.

**Fig. 1:**
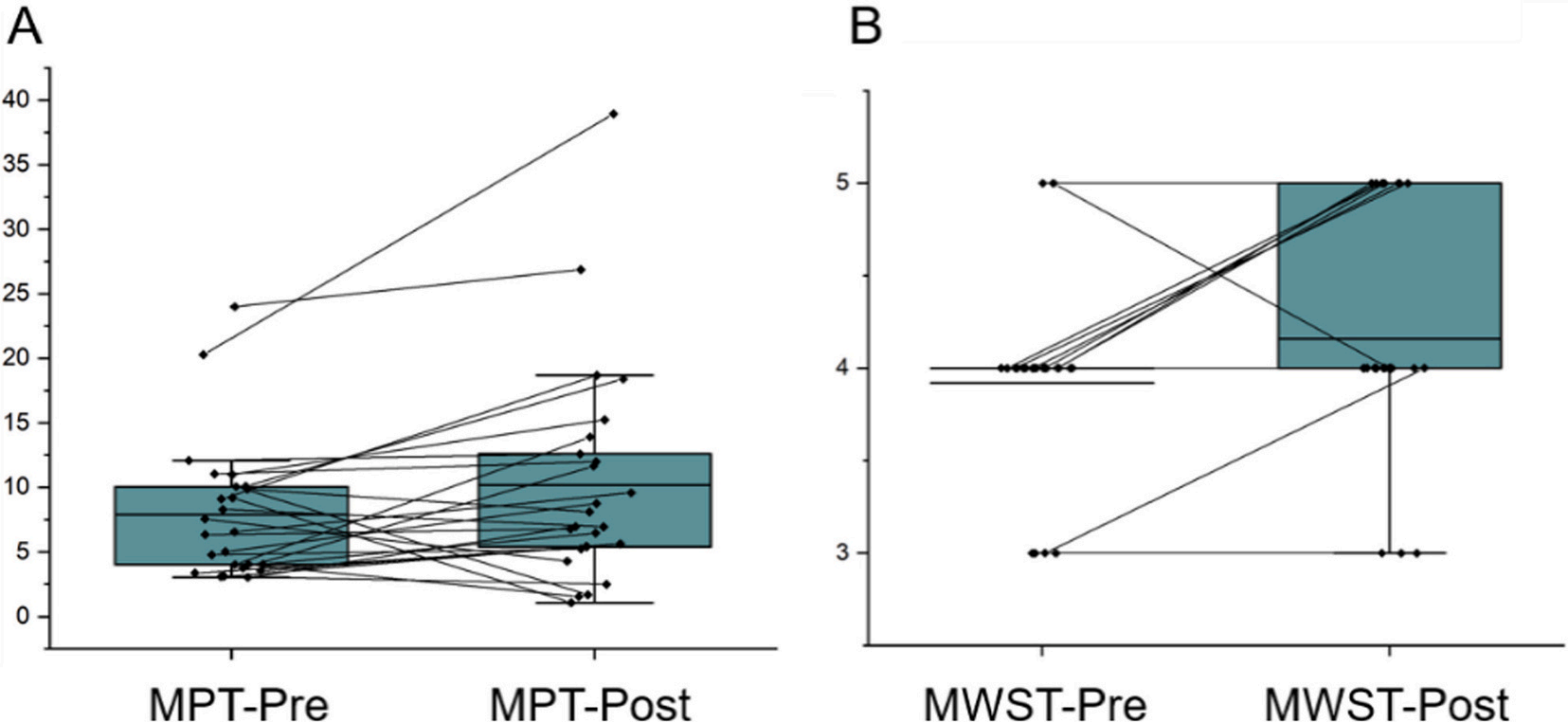
Exploratory pre–post changes in selected voice and swallowing outcomes. Paired individual data are shown for maximum phonation time (MPT) (A) and modified water swallowing test (MWST) score (B) before and after the 12-session group music recreation program added to stable background speech-language therapy. Each dot represents an individual participant, and paired observations from the same participant are connected by lines. Boxes indicate medians and interquartile ranges, and whiskers indicate ranges. Higher MPT and MWST scores indicate better performance. Statistical results, including Wilcoxon signed-rank p values, Bonferroni-adjusted *p* values, and effect sizes, are presented in Table 1. *n* = 25.

Changes in voice and swallowing outcomes before and after the 12-session group music recreation program are summarized in Table 1. In unadjusted Wilcoxon signed-rank tests, nominal pre–post changes were observed in maximum phonation time (MPT) and modified water swallowing test (MWST) (Fig. 1). MPT showed a median paired change of 2.23 s [95% CI, 0.47 to 3.72; *p* = 0.04; effect size *r* = 0.41], whereas MWST showed a median paired change of 0.00 [95% CI, 0.00 to 1.00; *p* = 0.03; effect size *r* = 0.42]. However, neither outcome remained statistically significant after Bonferroni correction.

**Table 1:** Changes in Voice and Swallowing Outcomes Before and After the 3-Month Rehabilitation Program.

Outcome	n	Pre-program median [IQR]	Post-program median [IQR]	Median paired change [95% CI]	Wilcoxon p	Bonferroni-adjusted p value	Effect size r
Maximum Phonation Time (seconds)	25	6.57 [3.90–10.08]	6.98 [5.32–13.25]	2.23 [0.47 to 3.72]	**0.04**	0.32	0.41
Mean Sound Pressure	25	85.10 [75.70–91.25]	90.30 [73.45–100.80]	3.10 [−4.70 to 8.20]	0.42	1.00	0.16
Vocal Handicap Index	25	35.00 [22.50–53.00]	30.00 [19.50–50.00]	−3.50 [−9.00 to 2.00]	0.07	0.55	0.36
Fujishima Dysphagia Severity Scale	25	3.00 [2.00–3.00]	2.00 [2.00–3.00]	0.00 [0.00 to 0.00]	0.27	1.00	0.22
Visual Assessment Scale for for swallowing	25	50.00 [31.00–77.00]	60.00 [39.50–80.00]	0.00 [−1.50 to 12.50]	0.39	1.00	0.17
Modified Water Swallowing Test	25	4.00 [4.00–4.00]	4.00 [4.00–5.00]	0.00 [0.00 to 1.00]	**0.03**	0.27	0.42
Repetitive Saliva Swallowing Test	25	3.00 [2.00–3.00]	3.00 [2.00–3.50]	0.00 [0.00 to 0.50]	0.31	1.00	0.20
Seirei Dysphagia Questionnaire	24	3.00 [1.00–9.75]	6.00 [1.25–10.25]	0.00 [−1.00 to 3.00]	0.24	1.00	0.24

VHI total scores showed a non-significant trend toward reduction, whereas mean sound pressure, Fujishima Dysphagia Severity Scale, VAS for swallowing difficulty, RSST, and Seirei Dysphagia Questionnaire did not show clear pre–post changes.

Overall, the program was feasible, and exploratory analyses suggested a favorable nominal signal for sustained phonation, particularly MPT. The nominal MWST finding should be interpreted cautiously because the median paired change was 0. No outcome remained statistically significant after correction for multiple comparisons.

## 4 Discussion

In this exploratory feasibility study of institutionalized patients with Parkinson's disease, a 12-session group music recreation program added to stable background speech-language therapy was feasible in a long-term care setting. Exploratory analyses showed nominal pre–post changes in selected outcomes, particularly MPT and MWST, although no outcome remained statistically significant after Bonferroni correction. These findings should therefore be interpreted as hypothesis-generating rather than evidence of efficacy.

With respect to swallowing, improvement was observed only in the modified water swallowing test. In contrast, the repetitive saliva swallowing test and the Dysphagia Severity Scale did not significantly change, and subjective swallowing ratings showed a non-significant trend toward worsening. Taken together, these findings indicate that swallowing did not globally improve. Rather, the rehabilitation may have enhanced task-specific swallowing performance under structured conditions without translating into broader functional or perceptual benefit. Although the magnitude of swallowing improvement was modest, even small physiological gains may be clinically relevant in frail institutionalized patients at high aspiration risk.

The nominal MWST finding may reflect changes in respiratory–laryngeal coordination, glottic closure, attention, or task familiarity during structured assessment. However, swallowing in daily life involves complex food consistencies, fatigue, posture, cognition, and environmental factors that are not captured by a simple water-swallowing task. In addition, pulmonary function was not assessed; therefore, we cannot determine whether the observed nominal changes were mediated by improved respiratory capacity, respiratory–phonatory coordination, or laryngeal-swallowing modulation.

Several limitations should be acknowledged. First, the uncontrolled, open-label pre–post design precludes causal inference. Second, multiple exploratory outcomes were examined, and no outcome remained statistically significant after Bonferroni correction; therefore, the nominal unadjusted findings should be interpreted cautiously. Third, baseline heterogeneity in age, disease duration, and disease severity may have influenced outcome changes. Because of the small sample size, the study was not designed to determine whether baseline characteristics modified the response to the intervention. Fourth, pulmonary function and instrumental swallowing assessments such as videofluoroscopy or fiberoptic endoscopic evaluation of swallowing were not performed. Fifth, outcome assessors were not blinded to the intervention period, and some assessments were conducted by personnel involved in routine clinical care; therefore, observer bias cannot be excluded. Finally, because of the small sample size and exploratory feasibility-oriented design, the study was not powered to evaluate dose-response relationships between session attendance and clinical outcomes.

In conclusion, adding a 12-session group music recreation program to stable background speech-language therapy was feasible in institutionalized patients with Parkinson's disease. Exploratory analyses showed nominal pre–post changes in selected voice and swallowing measures, particularly MPT, but no outcome remained statistically significant after correction for multiple comparisons. These findings should be regarded as hypothesis-generating and warrant confirmation in controlled studies.

## Financial disclosures

K.I. reports honoraria from Eisai Co., Ltd., FP Co., Ltd., AbbVie Inc., Grants-in-Aid for Scientific Research, and the Japan Agency for Medical Research and Development. H.A. is the founder of Shizuku Works, which provides music-based rehabilitation programs. Y·O, T.H., S·U, and A.Y. are employees of Super Court Co., Ltd., where the study was conducted. This study received no funding from Super Court Co., Ltd. S.T. declares no competing interests.

## Use of Artificial Intelligence Tools

Artificial intelligence–based language tools were used during manuscript preparation to assist with English language editing and clarity of expression. These tools were not used to generate scientific content, perform data analysis, or interpret results. All text was critically reviewed and edited by the authors, and all references and factual statements were verified against the original sources to ensure accuracy and prevent inclusion of fabricated information.

## Ethical Compliance Statement

The study protocol adhered to the principles of the Declaration of Helsinki and was approved by the Institutional Review Board of The University of Osaka (approval numbers 13,471 and 22,311). Written informed consent was obtained from all participants prior to enrollment. We confirm that we have read the Journal's position on issues involved in ethical publication and affirm that this work is consistent with those guidelines.

## CRediT authorship contribution statement

**Yasufumi Oi:** Writing – review & editing, Supervision, Methodology, Data curation, Conceptualization. **Haruna Aoki:** Writing – review & editing, Methodology, Investigation, Data curation, Conceptualization. **Tomoyuki Hatsuse:** Writing – review & editing, Supervision, Methodology, Data curation, Conceptualization. **Seira Taniguchi:** Writing – review & editing, Supervision, Methodology, Conceptualization. **Seiji Ueno:** Writing – review & editing, Supervision. **Akiyoshi Yamamoto:** Writing – review & editing, Supervision. **Kensuke Ikenaka:** Writing – review & editing, Writing – original draft, Validation, Resources, Methodology, Investigation, Formal analysis, Data curation, Conceptualization.

## Funding

This study was supported by JST FOREST Program, Grant Number JPMJFR231L. Potential conflicts of interest are disclosed below.

## Declaration of competing interest

The authors declare that they have no known competing financial interests or personal relationships that could have appeared to influence the work reported in this paper.

## References

[1] Lopez-Liria R., Parra-Egeda J., Vega-Ramirez F.A., Aguilar-Parra J.M., Trigueros-Ramos R., Morales-Gazquez M.J. (2020). Treatment of dysphagia in Parkinson’s disease: a systematic review. Int. J. Environ. Res. Public Health.

[2] Gandhi P., Steele C.M. (2022). Effectiveness of interventions for dysphagia in Parkinson disease: a systematic review. Am. J. Speech Lang. Pathol..

[3] Yeo M.S., Hwang J., Lee H.K., Kim S.J., Cho S.R. (2024). Therapeutic singing-induced swallowing exercise for dysphagia in advanced-stage Parkinson’s disease. Front. Neurol..

[4] Miles A., Jardine M., Johnston F., de Lisle M., Friary P., Allen J. (2017). Effect of Lee Silverman voice treatment (LSVT LOUD(R)) on swallowing and cough in Parkinson’s disease: a pilot study. J. Neurol. Sci..

[5] Taniguchi S., Marumoto K., Kajiyama Y., Revankar G., Inoue M., Yamamoto H. (2024). The validation of a Japanese version of the new freezing of gait questionnaire (NFOG-Q). Neurological Sci.: Official J. Italian Neurological Society and of the Italian Society of Clinical Neurophysiology..

[6] Ogawa T., Kajiyama Y., Ishido H., Chiba S., Revankar G.S., Nakano T. (2022). Decreased cerebrospinal fluid orexin levels not associated with clinical sleep disturbance in Parkinson’s disease: a retrospective study. PLoS One.

[7] Ikenaka K., Kajiyama Y., Aguirre C., Choong C.J., Taniguchi S., Doi J. (2024). Decreased hepatic enzymes reflect the decreased vitamin B6 levels in Parkinson’s disease patients. Pharmacol. Res. Perspect..

[8] Maslan J., Leng X., Rees C., Blalock D., Butler S.G. (2011). Maximum phonation time in healthy older adults. J. Voice.

[9] Svec J.G., Granqvist S. (2018). Tutorial and guidelines on measurement of sound pressure level in voice and speech. J. Speech Lang. Hear. Res..

[10] Portone C.R., Hapner E.R., McGregor L., Otto K., Johns M.M. (2007). Correlation of the voice handicap index (VHI) and the voice-related quality of life measure (V-RQOL). J. Voice.

[11] Tohara H., Saitoh E., Mays K.A., Kuhlemeier K., Palmer J.B. (2003). Three tests for predicting aspiration without videofluorography. Dysphagia.

[12] Fujishima I. (2003). Evaluation and management of dysphagia after stroke. Nihon Ronen Igakkai Zasshi..

